# Galectins and Carcinogenesis: Their Role in Head and Neck Carcinomas and Thyroid Carcinomas

**DOI:** 10.3390/ijms18122745

**Published:** 2017-12-18

**Authors:** Nadège Kindt, Fabrice Journe, Ghanem E. Ghanem, Sven Saussez

**Affiliations:** 1 Laboratory of Anatomy, Department of Human Anatomy and Experimental Oncology, Faculty of Medicine and Pharmacy, University of Mons (UMons), Pentagone 2A, 6 Ave du Champ de Mars, B-7000 Mons, Belgium; nadege.kindt@umons.ac.be (N.K.); fabrice.journe@umons.ac.be (F.J.); 2 Laboratory of Oncology and Experimental Surgery, Institut Jules Bordet, Université Libre de Bruxelles (ULB), 1000 Brussels, Belgium; gghanem@ulb.ac.be; 3 Department of Oto-Rhino-Laryngology, Université Libre de Bruxelles (ULB), CHU Saint-Pierre, 1000 Brussels, Belgium

**Keywords:** galectins, head and neck cancer, thyroid cancer

## Abstract

Head and neck cancers are among the most frequently occurring cancers worldwide. Of the molecular drivers described for these tumors, galectins play an important role via their interaction with several intracellular pathways. In this review, we will detail and discuss this role with specific reference to galectins-1, -3, and -7 in angiogenesis, cell proliferation, and invasion as well as in cell transformation and cancer progression. Furthermore, we will evaluate the prognostic value of galectin expression in head and neck cancers including those with oral cavity, salivary gland, and nasopharyngeal pathologies. In addition, we will discuss the involvement of these galectins in thyroid cancers where their altered expression is proposed as a new diagnostic biomarker.

## 1. Introduction

Head and neck cancers are among the most frequently occurring cancers worldwide, and squamous cell carcinoma is the predominant histological type [1]. Related major risk factors include tobacco and alcohol use; but in recent years, HPV infection has appeared as an additional risk factor that contributes to head and neck squamous cell carcinoma (HNSCC), although contradictory prognostic values have been reported [2,3]. This contradiction can be explained by the choice of the studied population. Indeed, two HPV-positive cancer populations may be distinguished. The first group is mainly composed of non-smoking younger adults with a favorable prognosis, while the second includes smoking and drinking patients with a poor prognosis [4,5].

The thyroid is an endocrine gland located in the lower part of the neck, and its malignancies are the most frequent endocrine cancers, with an increasing incidence over the last several years. Several types of thyroid carcinomas exist, including papillary carcinoma and follicular carcinoma, which are both well-differentiated and the most frequent histological thyroid cancers, as well as medullary carcinoma and anaplastic carcinoma, the latter being the most undifferentiated and aggressive. All differ in their incidence, prognosis and response to treatment [6]. The major risk factors of thyroid cancers are ionizing radiation exposure and pre-existent benign thyroid pathology, and recently obesity has been suggested as a risk factor [7].

**Figure 1:**
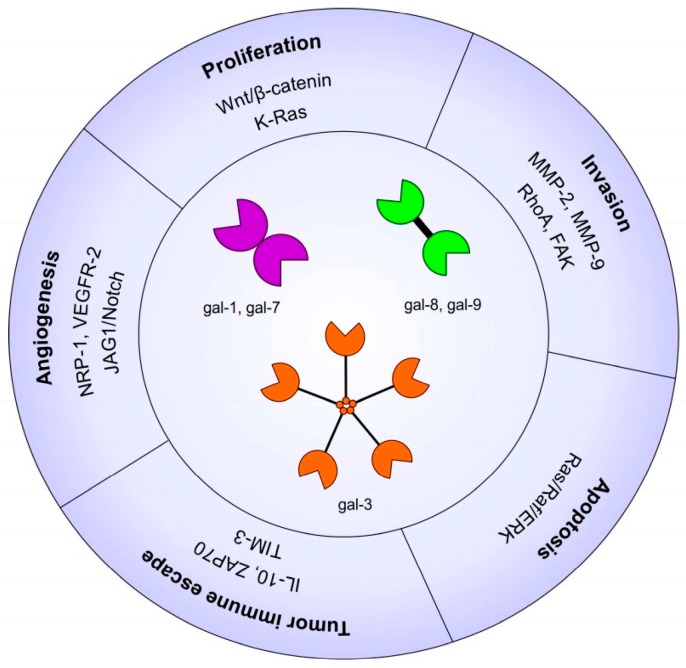
Hallmark of galectins in head and neck cancers. Gal-1, -3, -7, -8, and -9 in cancer progression affecting several pathways including proliferation, apoptosis, invasion, angiogenesis, and tumor-immune escape. Matrix Metalloproteinase (MMP); Interleukin (IL); Focal adhesion kinsase (FAK).

Galectins (gal) are proteins that are encoded by LGALS genes. They are members of the lectin family, of which 14 mammalian galectins have been identified. Galectins belong to the family of glycan-binding proteins and present three main structures (Figure 1). The first structure is the prototype galectin which is composed of one carbohydrate recognition domain (CRD) and can dimerize; this group is composed of gal-1, -2, -5, -7, -10, -11, -13, -14, and -15. The second structure is the tandem-repeat galectin which is composed of two CRD domains connected by a peptide bond; this group contains gal-4, -6, -8, -9, and -12. The third structure is a chimeric galectin with the unique gal-3 for which the CRD domain contains an N-terminal sequence that allows the oligomerization to form a pentamer. The galectins have an affinity for carbohydrates, including *N*-acetyl-lactosamine disaccharide (LacNac) or β-galactose and are found both in the intracellular and extracellular compartments. Moreover, galectins display various roles depending on their location. Indeed, in the intracellular compartment, gal-1 and -3 regulate some signaling pathways involved in cell survival by direct interaction with, notably, H-Ras and K-Ras pathways, respectively [8,9]. In the extracellular matrix, gal-3 contributes to the stimulation of endothelial cell migration and proliferation leading to angiogenesis promotion [10]. Thus, galectins are involved in several mechanisms leading and promoting cancer. The present review will focus on their implications in head and neck, as well as thyroid cancers.

## 2. Galectins in Head and Neck Cancer and Thyroid Cancer

Galectins contribute in many ways to carcinogenesis and tumor progression and are involved in biological processes such as cell growth and differentiation, cell adhesion, migration, invasion, and angiogenesis [10,11,12] (Figure 1). Gal-1 is more frequently studied in HNSCC whereas gal-3 is more extensively examined in thyroid cancer. In their respective cancers, these galectins are reported to be powerful diagnostic markers and are indicative of a good prognosis [13,14].

**Figure 2:**
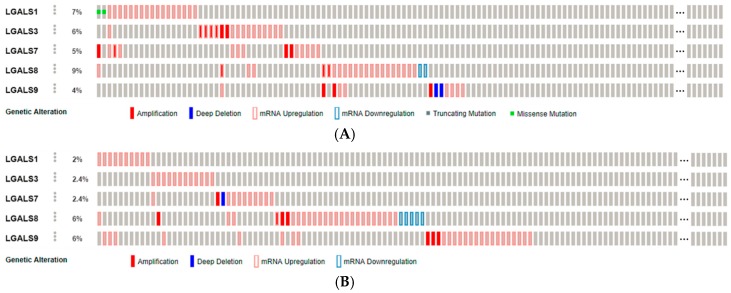
Alterations of galectin-1, -3, -7, -8, and -9 genes in head and neck squamous cell carcinoma (**A**) and thyroid cancer (**B**). Molecular alterations were investigated with the cBioPortal for Cancer Genomics [15], evaluating RNA expression, copy number variation, and mutation using The Cancer Genome Atlas (TCGA) datasets. Alteration events are displayed by samples ((**A**), *n* = 279; (**B**), *n* = 507).

In our review based on the most relevant data, gal-1, -3, -7, -8, and -9 are discussed in relation to head and neck and/or thyroid cancers. Genomic alterations of these galectins were investigated thanks to The Cancer Genome Atlas. The analysis of mRNA expression, copy number variation and mutation in 279 cases of head and neck squamous cell carcinomas using the cBioPortal for Cancer Genomics [15] revealed molecular alterations in only 7% of cases for gal-1, 6% for gal-3, 5% for gal-7, 9% for gal-8, and 4% for gal-9, mainly including mRNA up-regulation and a few gene amplification [16] (Figure 2A). The same analysis was done for 507 papillary thyroid carcinomas, and genomic alterations were reported in only 2% of cases for gal-1, 2.4% for gal-3, 2.4% for gal-7, 6% for gal-8, and 6% for gal-9, mainly involving mRNA up-regulation [17] (Figure 2B). Of note, no molecular alteration in these galectins was reported in the evaluation of 117 poorly-differentiated and anaplastic thyroid cancers [18].

### 2.1. Galectins in Carcinogenesis

HNSCCs are characterized by a field of cancerization around the main tumor. This tumor develops from normal epithelium to low and high-grade dysplasia which finally progresses to carcinoma. It is well known that most patients present several areas of dysplasia around the field of the main tumor. These other pre-neoplastic lesions could be the origin of additional head and neck carcinomas seen in the follow-up of the patient. Therefore, the study of the contribution of galectins in HNSCC carcinogenesis is very interesting. Regarding salivary gland transformation, it was demonstrated that the expression of gal-3 was strong in the cytoplasm of normal inter- and intra-lobular ductal cells and in benign lesions, while its expression was decreased in high grade carcinomas [19,20]. By contrast, expression of gal-1 was weaker than gal-3 in normal ductal cells with stronger labelling of myoepithelial cells, and in carcinomas gal-1 was expressed in the cytoplasm and/or the nucleus of the majority of tumors (Table 1) [19,20]. Gal-7 was expressed in the cytoplasm and the nuclei of normal ductal cells with a stronger immunoreactivity in the basal layer. In carcinoma, the expression of gal-7 decreased significantly compared to adenoma (Table 1) [20]. Finally, gal-8 was exclusively detected at high level in the cytoplasm of normal ductal cells while its expression decreased in cancer cells, with a labelling mainly localized in the cytoplasm and in some cases in the nucleus (Table 1) [20]. Concerning carcinoma of the oral cavity, gal-1 protein expression and mRNA level significantly increased during transformation (normal epithelium < dysplasia < carcinoma) [21,22,23]. Poorly differentiated carcinoma showed a stronger gal-1 expression compared to the well-differentiated one [21]. The expression of gal-3 protein and mRNA were also increased in carcinoma compared to normal epithelium (Table 1) [23,24]. Moreover, gal-1 and gal-3 serum levels were 8 and 3 fold higher, respectively, in oral cavity cancer patients compared to healthy volunteers [23]. Regarding laryngeal and hypopharyngeal carcinoma, it was established that gal-1 and gal-7 increased during carcinogenesis (Table 1), with a shift of gal-1 localization from the nucleus towards the cytoplasm during the progression of high grade dysplasia to carcinoma, and a shift of gal-7 from the cytoplasm to the nucleus between normal epithelium and dysplasia [25]. In nasopharyngeal cancer, it was reported that the gal-1 protein and mRNA expression increased in cancer tissue compared to normal tissue (Table 1) [26]. Additionally, Duray et al. observed that the expression of gal-9 increased in naso-sinusal carcinoma compared to non-malignant naso-sinusal diseases. In contrast, gal-8 expression decreased in naso-sinusal cancer compared to non-malignant naso-sinusal diseases (Table 1) [27].

Regarding thyroid cancer, multicenter studies reported that gal-3 expression increased in thyroid cancer tissues, definitively demonstrating its diagnostic value [28,29]. More recently, Arcolia et al. investigated gal-1 and gal-3 immunohistochemical expression in normal thyroid tissues, benign thyroid lesions and thyroid cancers, and reported that both galectin cytoplasmic immunostainings were significantly higher in cancer cells of malignant thyroid lesions compared to epithelial cells in benign lesions as well as in normal tissues [14]. These data complete a previous study showing that gal-3 expression was stronger in cancer tissue compared to non-malignant tissue (nodular goiter), while no difference was observed for gal-7 [30]. Additionally, gal-1 and -3 serum levels increased in carcinoma compared to healthy volunteers (Table 1) [31].

### 2.2. Galectins and Patient Prognosis

With a five-year relative survival rate of approximately 50%, patients with HNSCC have poor prognoses. The assumption that galectins could be used as biomarkers to identify HNSCC individuals with a better or a poorer prognosis is based on many arguments. Indeed, overexpression of gal-1 in HNSCC is associated with a poor prognosis in terms of recurrence-free survival and overall survival [32,33,34,35]. Moreover, it was demonstrated that patients with tongue carcinoma expressing low level of gal-3 had a favorable five-year disease-free survival (73.9%) compared to patients expressing high levels (40.8%) [36]. In adenoid cystic carcinoma, patients presenting an increase of gal-3 expression tended to have a shorter disease-free survival [37]. In addition, the combination of low gal-1 and gal-3 serum levels in HNSCC patients was significantly associated with a longer survival compared to HNSCC patients with high gal-1 or gal-3 [38]. Regarding gal-7, it was also demonstrated in stage IV hypopharyngeal carcinomas that high expression of gal-7 correlated with poor prognosis [39]. However, in laryngeal carcinoma, researchers have observed that a gal-3 positive tumor correlated with a better prognosis in terms of recurrence-free survival and overall survival and that gal-3 expression was positively associated with the histopathological grade and tumor keratinization [40,41]. Of note, molecular alterations, as evaluated using the cBioPortal for Cancer Genomics [15] in gal-1, -3, -7, -8, and -9, were not significantly associated with patient survival [16].

In thyroid cancer, well-differentiated thyroid carcinomas, including papillary and follicular carcinomas, are associated with an excellent prognosis, with 85% to 90% cure rates thanks to early detection and appropriate treatment. In contrast, undifferentiated anaplastic thyroid carcinoma is highly aggressive with a five-year survival rate of approximately 7% [42]. In this context, Kim et al. reported that the absence of gal-3 expression in papillary thyroid carcinomas was associated with pathological parameters considered less aggressive, including negative lymph node involvement [43]. However, another study indicated that gal-3 expression was not significantly associated with extra-thyroidal extension or lymph node metastases [44]. Additionally, genomic alterations in these galectins were not correlated with patient survival [17]. Thus, large-scale clinical studies are needed to verify the potential prognosis values of galectin expression in cancer associated with patient prognosis.

On the other hand, gal-3 and gal-1 have been suggested as diagnostic markers. Indeed, higher gal-3 expression correlated with the diagnosis of thyroid carcinoma [45,46], leading to the translation of gal-3 assessment in clinical settings [28,29], as well as the use of this lectin for thyroid cancer imaging in vivo by using an immunoPET targeting gal-3 to improve the diagnosis [47]. Our own recent findings demonstrated that gal-1 is also a useful immunohistochemical marker to discriminate malignant tumors from benign thyroid nodules [14]. Furthermore, we also validated that gal-3 is a sensitive marker for the diagnosis of thyroid malignancy, and we added support for its combination with CK19 and HBME-1 with the highest performance for the diagnosis of well-differentiated thyroid cancer [48]. By contrast, gal-7 and gal-8 were not differentially expressed between thyroid adenoma and cancer, suggesting that such galectins are not mainly involved in the carcinogenesis and tumor progression in HNSCC [48].

### 2.3. Galectins and Cell Proliferation/Survival

Galectins contribute to tumor growth by affecting cell proliferation, survival and apoptosis. They are present in the intracellular and extracellular compartments of the cells. Extracellularly, galectins can bind glycoproteins such as laminin, elastin, and fibronectin. Additionally, they can cross-link cell-surface glycoconjugates, which can activate signaling pathways within the cell leading to the modulation of cell cycle progression and apoptosis [49]. In the intracellular compartment, galectins also modulate cell cycle progression and apoptosis through various interactions with intracellular proteins implicated in these processes [49].

**Figure 3:**
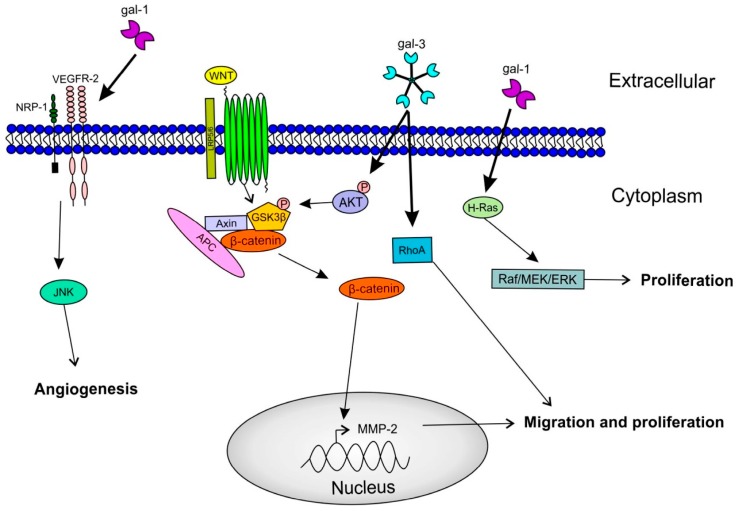
Involvement of galectins during carcinogenesis. Gal-1 interacts with Neuropilin-1/Vascular endothelial growth factor-2 (NPR-1/VEGFR-2) complex to promote endothelial cells migration through the c-Jun N-terminal kinase (JNK) pathway. Also, gal-1 interacts with H-Ras to stimulate cell proliferation by the stimulation of the Extracellular signal-regulated kinase (ERK) pathway. Gal-3 promotes cell proliferation and cell migration by the activation of β-catenin through the Phosphoinositide 3-kinase (PI3K)/Akt pathway, but also through Ras homolog family member A (RhoA) signaling. Glycogen synthase kinase 3β (GSK3β); Adenomatous polyposis coli (APC).

Gal-3 is the most studied galectin as to its involvement in tumor cell survival. Indeed, it shows anti-apoptotic properties; its translocation from the cytosol to mitochondria following exposure to pro-apoptotic stimuli blocks changes of the mitochondrial membrane potential, thereby preventing apoptosis [50]. Additionally, the mutation of gal-3 (serine^6^ to alanine or glutamic acid) prevents phosphorylation, resulting in a decrease of its anti-apoptotic activity [51]. Furthermore, gal-3 has a role in cell proliferation, as the inhibition of this protein by knockdown lead to the decrease of esophageal and renal cancer cell proliferation in vitro [52,53]. In oral tongue squamous cell carcinoma, an in vitro study using Tca8113 cell line established that the overexpression of gal-3 increased cell proliferation. Inversely, the knockdown of gal-3 in Tca8113 cells exhibited a decrease of cell proliferation. The latter study also showed that gal-3 acts on cell proliferation through the activation of the Wnt/β-catenin pathway (Figure 3) [24].

In thyroid cancer, it was demonstrated in vitro that gal-3 plays an anti-apoptotic role by its interaction with the pro-apoptotic Bax factor, which belongs to the Bcl-2 protein family [54]. Moreover, it is known that gal-3 interacts with K-Ras in cancer cells, leading to the activation of important signaling cascades such as PI3K/Akt [55]. Inhibition of gal-3 with Td131_1 demonstrated a synergistic effect with doxorubicin in a human ex vivo papillary thyroid cancer model by the activation of caspase 3 and PARP cleavage [56]. Furthermore, gal-3 inhibition with modified citrus pectin (MCP) combined with the inhibition of Ras with S-trans, trans-farnesyl thiosalicylic acid (FTS) not only drove the decrease of anaplastic cell proliferation both in vitro and in vivo, but also induced cell cycle arrest at the G1 phase and apoptosis of these cells [57]. 

Concerning gal-1, it was demonstrated that its knockdown caused a decrease in cell proliferation in anaplastic, but not in papillary thyroid cancer cells. In vivo, the orthotopic mouse model of anaplastic gal-1KD cells displayed a significant decrease in tumor growth [14]. Similar evidence was reported with ovarian cancer, and explained its effect on cell growth by the activation of the H-Ras/Raf/ERK pathway (Figure 3) [58]. Finally, the addition of recombinant gal-1 or gal-3 to the medium of three oral squamous cell carcinoma cell lines increased tumor cell proliferation [23].

### 2.4. Galectins and Cell Migration/Invasion

Tumor cell invasion contributes to the development of metastases. Several studies have brought to light the role of galectins in different metastatic processes, notably in cell adhesion, cell migration and invasiveness. Among all galectins, gal-3 is the most extensively studied regarding its role in metastasis occurrence. It interacts with adhesion molecules (CAM), such as integrins and cadherins, at the cell surface to promote cancer cell invasion [59]. For example, in pancreatic cancer cell lines, gal-3 silencing lead to a two-fold decreased in cell migration and invasion [60]. The mechanism by which gal-3 affects cell migration proceeds through an inhibition of the phosphorylation of Akt and GSK-3β occurring after 12 h, followed by a decrease of β-catenin expression after 48 hours. Indeed, the down-regulation of β-catenin leads to a lower expression of matrix metalloproteinase MMP-2, meaning that gal-3 may regulate the Wnt/β-catenin signaling pathway in cancer cells (Figure 3) [60]. Similar results have been published for HNSCC, where silencing of Wnt reduced the ability of gal-3 to stimulate cell migration and invasion of oral tongue squamous cell carcinoma [24].

In thyroid cancer, Shankar et al. observed that gal-3 and caveolin-1 (known to promote tumor cell migration [61]), are both up-regulated in differentiated thyroid cancer compared to benign lesions, and cause RhoA activation and FAK stabilization in focal adhesions, ultimately leading to cell migration [62]. Interestingly, the knockdown of RhoA in tongue squamous cell carcinoma (TSCC) cells negatively affects cell migration and invasion [63]. Moreover, RhoA protein was expressed at higher levels in TSCC xenografts in mice with lymph node metastases. Silencing of RhoA in such tumors downregulates the expression of gal-3, β-catenin and MMP-9 (Figure 3) [63].

Concerning gal-1, it was demonstrated that in cancer-associated fibroblasts (CAF), which play an important role in cell migration and invasion, under-expression of gal-1 significantly decreased breast cancer cell migration. Additionally, MMP-9 expression levels were attenuated in CAF silenced for gal-1, thus limiting the degradation of the extracellular matrix [64]. Similarly, in oral squamous cell carcinoma (OSCC), the silencing of gal-1 in CAF negatively affected cell migration and invasion by reducing the amount of monocyte chemotactic protein-1 (MCP-1/CCL2) [65]. Moreover, grafting OSCC cells mixed with CAF in mice allowed a faster tumor growth than OSCC cells alone, but also a fewer number of circulating tumor cells when gal-1 is under-expressed in CAF [65]. Another study by Wu et al. reported that overexpression of gal-1 in an OSCC cell line leads to an increase of MMP-2 and MMP-9 mRNA in vitro [66]. Additional data suggest that gal-1 highly affects actin filament localization, with modulation of filopodia formation, and stimulates tumor cell adhesion, migration, and invasiveness [66,67]. In vivo, Wu et al. demonstrated that gal-1 overexpression in oral cancer cells was associated with a higher number of lung metastases [66]. In thyroid cancer, our group recently demonstrated that the knockdown of gal-1 in papillary and anaplastic thyroid cancer cell lines induced decreased cell motility and invasion [14].

### 2.5. Galectins and Angiogenesis

Angiogenesis is an important event in cancer progression, promoting tumor growth and metastasis. Gal-1 and gal-3 are the two main galectins studied in angiogenesis in relation to cell proliferation and invasion. It has been reported that activation of endothelial cells in vitro induced an increase of gal-1 expression in the extracellular compartment [68]. In vivo, gal-1 null mice showing no expression of gal-1 in endothelial cells had a lower number of vessels in the tumor site compared to wild type mice expressing high gal-1 levels [69]. In oral cavity cancers, gal-1 is overexpressed in cancer associated-endothelial cells compared to normal endothelial cells [70]. Moreover, human umbilical vein endothelial cell (HUVECs) adhesion to laminin was significantly increased by stimulating the expression of extracellular gal-1, and dramatically decreased by its silencing [70].

Additionally, gal-1 binds the neuropilin-1 (NRP-1) on endothelial cells. NRP-1 is an important mediator of angiogenesis that can facilitate the binding of the vascular endothelial growth factor A (VEGF-A) to VEGFR-2, enhancing the phosphorylation of the receptor and the activation of angiogenesis. VEGFR-2 silencing leads to a reduction in gal-1-induced HUVECs migration. This activation of endothelial cell migration by gal-1 via the complex of VEGFR-2/NRP-1 required the participation of the JNK pathway (Figure 3) [70]. D’Haene et al. also showed that gal-1 and gal-3 might mediate angiogenesis by causing increased VEGF receptor density on endothelial cells, making them more accessible to low levels of endogenous VEGF [71].

Concerning gal-3, Chen et al. demonstrated that gal-3 was able to induce the release of pro-inflammatory cytokine such as IL-6, G-CSF, GM-CSF, and sICAM-1 from endothelial cells, which lead to the promotion of metastasis by increasing endothelial cell surface adhesion molecules, such as integrin αvβ1. Cytokine release might be regulated by the binding of gal-3 to the transmembrane protein MUC-1, because MUC-1 negative cells do not abundantly release such cytokines [72]. Additionally, endogenous gal-3 silencing significantly reduced VEGF- and bFGF-mediated endothelial cell migration, as well as capillary tubule formation in vitro, with a reversible effect when exogenous gal-3 was added to the cells [73]. Finally, gal-3 also induced angiogenesis through JAG1/Notch pathway activation, leading to endothelial cell sprouting [74]. 

However, regarding head and neck and thyroid cancers, there is a lack of data concerning the role of galectins in angiogenesis.

### 2.6. Galectins and Tumor Immune Escape

Some members of the glycan-binding protein family, such as gal-1, -3, and -9, are promoters of tumor immune evasion. Indeed, gal-1 is reported to induce growth arrest and apoptosis of activated T cells [75]. The mechanism of apoptosis induced by tumor cell-derived gal-1 could be due to the interaction of gal-1 with several surface glycoproteins of T cells such as CD45, CD43, CD7, and CD3 [76,77]. Furthermore, Kovacs et al. showed that gal-1 leads to T cell apoptosis through the stimulation of the tyrosine kinases p56lck and ZAP70, depolarization of the mitochondrial membrane, and activation of the caspase cascade [78]. Additionally, gal-1 is able to promote the production of IL-10 and to decrease the secretion of IFN-γ, together contributing to the immune-tolerance of tumor cells [79]. In head and neck cancers, more particularly in gingival carcinoma, it was demonstrated that cancer tissue highly expressing gal-1 correlated with a decrease in T cell infiltration and an increase in CD3+ and CD8+ T cell apoptosis [35].

On the other hand, it was previously documented that extracellular gal-3 was able to induce apoptosis of lymphoid cells, human PBMCs, and activated mouse T cells after binding to the cell surface [80]. Peng et al. also demonstrated that soluble gal-3 inhibited T cell responses in vivo [81]. Furthermore, several studies showed that gal-3 drove to the loss of natural killer (NK) cell action. Indeed, binding of gal-3 to poly-*N*-acetyllactosamine residue of cell surface mucin (MUC1) attenuated the interaction of tumor cells with NK cells, leading to tumor cell evasion from NK cell immunity [82]. Also, the interaction of gal-3 with NKp30 receptors on NK cells inhibited NK-mediated cytolysis. Moreover, the down-regulation of gal-3 in HeLa cells increased the sensitivity of tumor cells to lysis by NK cells [83]. 

Once again, there is no specific data concerning gal-3 in tumor immune evasion in head and neck or thyroid cancers.

Finally, gal-9 was identified, by mass spectrometry in 2005, as a ligand of immunoglobulins and mucin-domain-containing molecule-3 (TIM-3) that is specifically expressed in IFN-γ-producing T cells [84]. It was demonstrated that this binding can lead to the death of Th1 cells but can also inhibit Th17 polarization and advance the expansion of FoxP3+ Tregs [84,85]. In thyroid cancer, Severson et al. showed that gal-9 is expressed by tumor-infiltrating lymph nodes, and thus may contribute to immune dysfunction, suggesting that immune-modulating therapies that inhibit TIM-3/gal-9 may be viable options for patients with advanced diseases [86].

### 2.7. Clinical Potential of Galectin Inhibition

Several preclinical and clinical studies in phase I and II have used different kinds of galectin inhibitors, such as GM-CT-01 and GR-MD-02 in lymphoma, breast cancer, and melanoma. GM-CT-01 (DANAVAT^®^) is a galactomannan derived from plants which binds to gal-1. In vivo data revealed that injection of GM-CT-01 to colon tumor-bearing mice potentiated the anti-tumor activity of 5-fluorouracil (5-FU) [87]. In metastatic colon cancer patients, phase I and II demonstrated that the combination of GM-CT-01 with 5-FU increased the longevity of patients by 46% and decreased the serious adverse effects by 41% [87]. GM-CT-01 was also studied as a booster of tumor-infiltrating lymphocyte (TIL) function; this action is due to an increase in the release of IFNγ, allowing tumor regression [88]. This property is used in a phase I/II study in which GM-CT-01 is associated with a peptide vaccination to induce a more efficient and long-lasting anti-tumor immune response in metastatic melanoma patients (NCT01723813). Another inhibitor tested in pre-clinical and clinical studies is GR-MD-02, which binds to the carbohydrate-binding domain of gal-3. This inhibitor was developed by Galectins Therapeutics (Norcross, GA, USA) and was used in mice models of melanoma, breast cancer, prostate cancer, and sarcoma. The researchers observed in these models that GR-MD-02 potentiated the effect of immune modulators such as anti-PD1, anti-CTLA4, and anti-OX40 antibodies by reducing tumor size and improving mice survival [88]. Finally, clinical studies combined GR-MD-02 with other therapeutic molecules, such as ipilimumab (anti-CTLA4 antibody) in metastatic melanoma patients (NCT02117362) and pembrolizumab (anti-PD-1 antibody) in patients with metastatic melanoma, non-small cell lung cancer, and head and neck cancer (NCT02575404). In the latter study, one partial response has been observed showing a marked reduction in tumor size at week 12 of therapy, after 3 doses of GR-MD-02 and pembrolizumab combination [89].

## 3. Conclusions

Galectins, especially gal-1 and gal-3, have been largely studied in head and neck carcinoma and thyroid cancer (Table 2). They participate in carcinogenesis and tumor progression by stimulating cancer cell proliferation and survival, migration and invasion, and angiogenesis, as well as immune system escape through multiple mechanisms. Regarding their clinical value, the most recent studies revealed that galectins may be used as markers for prognosis in head and neck cancer and for diagnosis of thyroid carcinoma. Indeed, the use of gal-3 thyrotest preoperatively can help to characterize benign from malignant lesions of the thyroid, which could prevent unnecessary thyroid surgical procedures. However, larger clinical trials are required to validate these findings in head and neck cancer. Of note, the number of genomic alterations was very low in galectins and did not correlate with patient survival. Globally, these data also support galectins as potential targets for anti-cancer therapy. Several in vitro studies are promising and a few clinical trials are ongoing. Similarly, galectin inhibitors/antagonists can also be good options, and deserve to be tested in head and neck carcinoma and thyroid cancers, most likely in combination with various other therapies. 

## Figures and Tables

**Table 1:** Galectin-1, -3, -7, -8, and -9 expression during carcinogenesis of head and neck and thyroid carcinomas.

Type of Tissues	Gal-1	Gal-3	Gal-7	Gal-8	Gal-9	Ref.
Salivary gland carcinomas	* 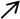	** 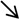	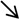	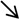		[19,20]
Oral cavity carcinoma	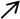	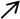				[21,22,23,24]
Laryngeal carcinoma	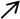		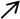			[25]
Hypopharyngeal carcinoma	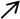		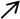			[25]
Naso-pharyngeal carcinoma	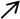			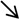	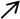	[26,27]
Thyroid carcinomas	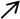	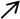	No change			[14,28,29,30,31]

* Increase; ** Decrease.

**Table 2:** Main references supporting a role for galectins in head and neck and thyroid cancer cell proliferation, migration/invasion, angiogenesis, and tumor immune escape.

	Gal-1		Gal-3		Gal-9	
	HNSCC	TC	HNSCC	TC	HNSCC	TC
Cell proliferation	[22]	[14]	[23]	[54,56]		
Apoptosis				[53,55,56]		
Cell migration/invasion	[64,65]	[14]	[23,62]	[61]		
Angiogenesis	[69]					
Tumor immune escape	[34]					[85]

HNSCC: Head and neck squamous cell carcinoma; TC: Thyroid carcinoma.
